# Methylation at cg05575921 of a smoking-related gene (AHRR) in non-smoking Taiwanese adults residing in areas with different PM_2.5_ concentrations

**DOI:** 10.1186/s13148-019-0662-9

**Published:** 2019-05-06

**Authors:** Disline Manli Tantoh, Kuan-Jung Lee, Oswald Ndi Nfor, Yi-Chia Liaw, Chin Lin, Hou-Wei Chu, Pei-Hsin Chen, Shu-Yi Hsu, Wen-Hsiu Liu, Chen-Chang Ho, Chia-Chi Lung, Ming-Fang Wu, Yi-Ching Liaw, Tonmoy Debnath, Yung-Po Liaw

**Affiliations:** 1 Department of Public Health and Institute of Public Health, Chung Shan Medical University, No. 110 Sec. 1 Jianguo N. Road, Taichung City, 40201 Taiwan; 2 Department of Medical Education, Taipei Veterans General Hospital, Taipei, Taiwan; 3 Graduate Institute of Life Sciences, National Defense Medical Center, Taipei, Taiwan; 4 Institute of Biomedical Sciences, Academia Sinica, Taipei, Taiwan; 5 Department of Physical Education, Fu-Jen Catholic University, New Taipei City, Taiwan; 6 School of Medicine, Chung Shan Medical University, Taichung City, Taiwan; 7 Institute of Clinical Medicine, National Yang-Ming University, Taipei, Taiwan; 8 Department of Family and Community Medicine, Chung Shan Medical University Hospital, Taichung City, Taiwan

**Keywords:** epigenetics, non-smoking, air pollution, residential area, AHRR, Taiwan Biobank

## Abstract

**Background:**

DNA methylation is associated with cancer, metabolic, neurological, and autoimmune disorders. Hypomethylation of aryl hydrocarbon receptor repressor (AHRR) especially at cg05575921 is associated with smoking and lung cancer. Studies on the association between AHRR methylation at cg05575921 and sources of polycyclic aromatic hydrocarbon (PAH) other than smoking are limited. The aim of our study was to assess the pattern of blood DNA methylation at cg05575921 in non-smoking Taiwanese adults living in areas with different PM_2.5_ levels.

**Methods:**

Data on blood DNA methylation, smoking, and residence were retrieved from the Taiwan Biobank dataset (2008–2015). Current and former smokers, as well as individuals with incomplete information were excluded from the current study. The final analysis included 708 participants (279 men and 429 women) aged 30–70 years. PM_2.5_ levels have been shown to increase as one moves from the northern through central towards southern Taiwan. Based on this trend, the study areas were categorized into northern, north-central, central, and southern regions.

**Results:**

Living in PM_2.5_ areas was associated with lower methylation levels: compared with the northern area (reference area), living in north-central, central, and southern areas was associated with lower methylation levels at cg05575921. However, only methylation levels in those living in central and southern areas were significant (*β* **=** − 0.01003, *P* = 0.009 and *β* = − 0.01480, *P* < 0.001, respectively. Even though methylation levels in those living in the north-central area were not statistically significant, the test for linear trend was significant (*P* < 0.001). When PM_2.5_ was included in the regression model, a unit increase in PM_2.5_ was associated with 0.00115 (*P* < 0.001) lower cg05575921 methylation levels.

**Conclusion:**

Living in PM_2.5_ areas was inversely associated with blood AHRR methylation levels at cg05575921. The methylation levels were lowest in participants residing in southern followed by central and north-central areas. Moreover, when PM_2.5_ was included in the regression model, it was inversely associated with methylation levels at cg05575921. Blood methylation at cg05575921 (AHRR) in non-smokers might indicate different exposures to PM_2.5_ and lung cancer which is a PM_2.5_-related disease.

**Electronic supplementary material:**

The online version of this article (10.1186/s13148-019-0662-9) contains supplementary material, which is available to authorized users.

## Background

DNA methylation, an epigenetic process characterized by the addition of a methyl group to a DNA molecule influences the expression of genes [1, 2]. It is associated with several health conditions including cancer, metabolic, neurological, psychiatric, and autoimmune disorders [1–5]. A greater portion of DNA methylation in humans takes place in regions called CG or CpG sites, where a cytosine precedes a guanine nucleotide [1, 2]. Due to its epigenetic nature, DNA methylation is associated with several environmental factors including cigarette smoking, exercise, and alcohol drinking, among others [6, 7].

The gene, aryl hydrocarbon receptor repressor (AHRR), mediates the metabolism of xenobiotic particles like toxic cigarette smoke components [8, 9]. This gene is located on chromosome 5 which is believed to possess several tumor suppressor genes [10, 11]. Blood AHRR methylation (especially at cg0557592) is inversely associated with both smoking [4, 5, 8, 9, 12–16] and lung cancer [4, 12, 13].

Cigarette smoke and PM_2.5_ are both inhalable carcinogenic factors composed of a complex mixture of chemicals, one of which is polycyclic aromatic hydrocarbons (PAHs) [11, 17–19]. PM-related PAHs pose most of the health problems in humans [20]. Exposure to PAHs is capable of activating the aryl hydrocarbon receptor (AHR) [17, 21] and is thought to be responsible for variations in smoking-related AHRR methylation [9]. Even though AHR activation is associated with particulate matter [22, 23], research on the association between methylation at cg05575921 (AHRR) and PM_2.5_ is limited. In a cross-sectional study, AHRR (cg05575921) hypomethylation in non-smokers was associated with exposure to second-hand smoke (SHS) but not PM_2.5_ [24]. Moreover, in a recent review on DNA methylation and environmental factors, several studies were listed to have demonstrated associations between AHRR hypomethylation and tobacco smoke. However, none was listed to have demonstrated an association between AHRR hypomethylation and exposure to air pollution or PAHs [25].

In a study conducted in Taiwan, PM_2.5_ was used as a proxy marker for PAHs [20]. PM_2.5_ concentrations in northern Taiwan are lower than in central and southern areas [26–29]. Because PAHs are present in both PM_2.5_ and cigarette smoke, AHRR (cg05575921) methylation in smokers might be comparable with that in non-smokers exposed to PM_2.5_. Therefore, we used an epigenetic approach to investigate the pattern of blood methylation at cg05575921 in non-smoking Taiwanese adults residing in areas with different PM_2.5_ concentrations.

## Results

Table 1 shows the general characteristics of study participants. There were 708 participants comprising 279 men (mean age = 49.42 ± 11.76 years) and 429 women (mean age = 49.49 ± 10.97 years). The mean (± SD) methylation level at cg05575921 was 0.83604 ± 0.00154. In general, 244, 121, 134, and 209 participants resided in the northern, north-central, central, and southern areas, respectively. Table 2 shows the mean (± SD) concentrations of PM_2.5_ (μg/m^3^) from 2006 to 2011 in the study areas. These concentrations were 27.32 ± 4.34, 28.65 ± 2.13, 35.72 ± 3.75, and 39.81 ± 2.10 μg/m^3^ for the northern, north-central, central, and southern areas, respectively. Table 3 shows the association between living in PM_2.5_ areas and AHRR (cg05575921) methylation. With the northern area as the reference, residing in north-central, central, and southern areas was associated with lower blood methylation levels at cg05575921. When SHS was included in the analysis, the regression coefficients (*β*) were − 0.00274 (*P* = 0.503), − 0.01003 (*P* = 0.009), and − 0.01480 (*P* < 0.001), respectively (Table 3, model 1). That is, the blood methylation levels in participants residing in north-central, central, and southern areas were lower when compared to those in the northern area. The differences were − 0.00274, − 0.01003, and − 0.01480, respectively. After SHS was excluded from the analysis, the regression coefficients (*β*) were − 0.00028 (*P* = 0.947), − 0.01069 (*P* = 0.009), and − 0.01487 (*P* < 0.001), respectively (Table 3, model 2). Even though methylation levels in participants who lived in north-central areas were not statistically significant, the test for linear trend was statistically significant (*P* trend < 0.001) in both models (Table 3, models 1 and 2). The mean PM_2.5_ concentration from 2006–2011 was significantly associated with lower blood AHRR methylation levels at cg05575921 (Table 4). A unit increase in PM_2.5_ concentration was associated with 0.00115 (*P* < 0.001) lower methylation when SHS was included in the analysis (Table 4, model 1). Similarly, a unit increase in PM_2.5_ concentration was associated with 0.00124 (*P* < 0.001) lower methylation after SHS was excluded from the analysis (Table 4, model 2). Spearman analysis showed a significant negative correlation (β = − 0.78329; *P* < 0.001) between PM_2.5_ concentration (μg/m^3^) and mean methylation levels (Additional file 1). The associations between PM_2.5_ and 176 sites in the AHRR promoter region are shown in Additional file 2. In addition to cg05575921, some other sites that were significantly associated with PM_2.5_ include cg26703534 (*β* = − 0.00127; *P* < 0.001), cg25648203 (*β* = − 0.00078; *P* < 0.001), and cg21161138 (*β* = − 0.00046; *P* = 0.007).

**Table 1:** General characteristics of study participants (2008–2015)

Variable	Men	Women		All participants
	*n* = 279	*n* = 429	*P* value	*n* = 708
cg05575921 (*β* value)	0.84260 ± 0.03740	0.82650 ± 0.03920	< .001	0.83626 ± 0.03892
Area			0.833	
Northern	92 (32.97%)	152 (35.43%)		244 (34.46%)
North-Central	46 (16.49%)	75 (17.48%)		121 (17.09%)
Central	54 (19.35%)	80 (18.65%)		134 (18.93%)
Southern	87 (31.18%)	122 (28.44%)		209 (29.52%)
Age (years)	49.42 ± 11.76	49.49 ± 10.97	0.926	49.46 ± 11.28
Alcohol Drinking			< .001	
No	251 (89.96%)	423 (98.60%)		674 (95.20%)
Former	7 (2.51%)	3 (0.70%)		10 (1.41%)
Current	21 (7.53%)	3 (0.70%)		24 (3.39%)
Exercise				
No	157 (56.27%)	240 (55.94%)	0.931	397 (56.07%)
Yes	122 (43.73%)	189 (44.06)		311 (43.93%)
BMI (kg/m^2^)	23.52 ± 3.57	24.95 ± 3.44	< .001	24.08 ± 3.59
SHS			0.598	
No	251 (89.96%)	391 (91.14%)		642 (90.68%)
Yes	28 (10.04%)	38 (8.86%)		66 (9.32%)

Continuous variables are presented as mean ± SD while categorical variables are presented as numbers (%)

*SD* standard deviation, *SHS* second-hand smoke

**Table 2:** Mean (± SD) concentrations of PM_2.5_ (μg/m3) from 2006–2011 in the study areas

Area	*n*	2006–2011	2006	2007	2008	2009	2010	2011
Northern	18	27.32 ± 4.34	27.43 ± 5.03	28.69 ± 5.39	27.33 ± 5.48	25.96 ± 5.32	25.98 ± 5.37	26.61 ± 5.30
North-Central	12	28.65 ± 2.13	29.81 ± 2.34	29.39 ± 2.98	29.75 ± 2.59	28.74 ± 2.47	26.93 ± 1.82	27.28 ± 2.84
Central	16	35.72 ± 3.75	36.27 ± 3.20	36.64 ± 3.81	35.56 ± 3.76	37.67 ± 3.63	34.83 ± 3.02	36.17 ± 5.34
Southern	7	39.81 ± 2.10	40.75 ± 2.30	41.00 ± 2.91	41.62 ± 3.37	40.08 ± 3.41	36.81 ± 3.15	38.05 ± 4.04

*SD* standard deviation, *n* number of monitoring stations

**Table 3:** Multiple linear regression showing the association between living in PM_2.5_ areas and AHRR (cg05575921) methylation

Variable	Model 1		Model 2		
	*β*	*P* value	*β*	*P* value	
Area (reference: Northern)	–	–	–	–	
North-Central	− 0.00274	0.503	− 0.0028	0.947	
Central	− 0.01003	0.009	− 0.01069	0.009	
Southern	− 0.01480	< .001	− 0.01487	< .001	
*P* trend	< .001			< .001	
Sex (reference: women)					
Men	− 0.01491	< .001	− 0.01413	< .001	
Age	− 0.00023	0.085	− 0.00022	0.114	
Alcohol Drinking (reference: no)					
Former	− 0.01041	0.358	− 0.01497	0.238	
Current	− 0.00532	0.475	− 0.00424	0.609	
Exercise (reference: no)					
Yes	− 0.00199	0.490	− 0.00180	0.552	
BMI	− 0.00009	0.819	− 0.00005	0.906	
SHS (reference: no)			–	–	
Yes	− 0.00119	0.796	–	–	

*Model 1* included SHS in the analysis, *model 2* excluded SHS from the analysis, *SHS* second-hand smoke

**Table 4:** Multiple linear regression showing the association between mean PM_2.5_ (μg/m3) from 2006–2011 and AHRR (cg05575921) methylation

Variable	Model 1		Model 2	
	*β*	*P* value	*β*	*P* value
PM_2.5_	− 0.00115	< .001	− 0.00124	< .001
Sex (reference: women)				
Men	− 0.01489	< .001	− 0.01416	< .001
Age	− 0.00023	0.083	− 0.00022	0.116
Alcohol Drinking (reference: no)				
Former	− 0.01043	0.357	− 0.01494	0.238
Current	− 0.00534	0.473	− 0.00430	0.603
Exercise (reference: no)				
Yes	− 0.00196	0.495	− 0.00183	0.545
BMI	− 0.00007	0.844	0.00003	0.942
SHS (reference: no)			–	–
Yes	− 0.00115	0.802	–	–

*Model 1* included SHS in the analysis, *model 2* excluded SHS from the analysis, *SHS* second-hand smoke

## Discussion

Studies on the association between AHRR methylation and sources of polycyclic aromatic hydrocarbon (PAH) other than smoking are limited. To our knowledge, the current study is the first to assess blood AHRR methylation in non-smokers residing in areas with different concentrations of PM_2.5_. In general, PM_2.5_ was inversely associated with blood AHRR (cg05575921) methylation. Compared with the northern area, living in north-central, central, and southern areas was associated with lower blood AHRR methylation levels at cg05575921.

Living in areas with higher PM_2.5_ concentrations has been associated with greater exposure to PAH [20]. Particulate matter-related PAHs have been reported as potential activators of AHR [17]. PM_2.5_ and cigarette smoke both contain PAHs which can induce AHRR methylation [11]. Moreover, they are both inhalable and carcinogenic [19]. Several studies have explored the association between PM_2.5_ and DNA methylation in blood [23, 30] and placenta [31]. Nonetheless, emphasis has not been laid on blood AHRR methylation in non-smokers. In light of this, we investigated the association between living in PM_2.5_ areas and AHRR methylation at cg05575921 in non-smokers. We adjusted for exposure to SHS since significant inverse associations have been found between methylation at cg05575921 and exposure to SHS in non-smokers [24].

Cg05575921 was selected because it has been shown to be highly sensitive and specific in identifying and distinguishing smoking status as well as tracking trends in smoking termination and reduction [12, 15, 32–34]. For instance, current smokers showed the highest hypomethylation compared to former and or non-smokers [4, 5, 13–16]. Moreover, this methylation site has been shown to be the most statistically significant smoking-related site in several original investigations [13, 16, 35–38] and a meta-analysis [39].

In our study, it was hypothesized that blood methylation patterns at cg05575921 (AHRR) in non-smokers residing in areas with different PM_2.5_ concentrations might be similar to those observed based on smoking status. As expected, the results were similar to those from several studies that were conducted among non-, former, and current smokers [4, 5, 13–16]. That is, blood methylation levels at cg05575921 in non-smokers residing in Southern Taiwan were lowest followed by Central and Northern Taiwan with significant linear trends. The regression coefficients were − 0.00028, − 0.01069, and − 0.01487 for north-central, central, and southern areas, respectively. Previous studies have reported regression coefficients of 0.83, 0.79, and 0.59 for never, former, and current smokers [5] and 0.878, 0.829, and 0.772 for non-, light, and heavy smokers, respectively [16]. Moreover, the methylation extent was 64, 60, and 50 % among never, former, and current smokers [4]. PM_2.5_ levels are higher in Southern and Central compared to Northern Taiwan [26–29]. There are many heavy industries (e.g., petrochemical plants) in these areas [27, 28, 40]. Emissions from these industries are believed to be one of the main sources of PM_2.5_- and PM_2.5_-bound PAHs [41–43]. Higher concentrations of PM_2.5_-bound PAHs have also been reported in the industrial cities of Christchurch and Guadalajara in New Zealand [43, 44] and Mexico [43, 45].

Some of the merits of our study include (1) the relatively larger sample size, (2) the adjustment for exposure to SHS to avoid its confounding effect, and (3) stratification of participants into four areas known for different PM_2.5_ levels. However, the study is limited in that DNA methylation was determined using the Illumina Infinium MethylationEPIC BeadChip which covers 850,000 methylation sites. Even though this microarray has been validated as a very reliable genomic platform for determining DNA methylation patterns in the human genome [46], validation experiments were not performed to confirm our findings. Therefore, future investigations to support our results are recommended. Moreover, functional correlation between cg05575921 methylation and AHRR mRNA gene expression was not evaluated in this study. Furthermore, the actual concentrations of PM_2.5_ in individuals could not be determined since there are no validated tools for individual exposure estimates. Air quality indices from nearby monitoring stations are usually used for air pollution estimation [47]. In the current study, the number of monitoring stations corresponding to the participants’ residence was relatively small. This is because some counties involved in the study do not have monitoring stations; hence, PM_2.5_ estimates were not available for participants living in such areas. However, we think our results are plausible because variations in PM_2.5_ concentration in various areas in Taiwan are well known [26–29]. Moreover, the inclusion of mean PM_2.5_ concentration from the study areas (2006–2011) into our regression model showed significant inverse associations between PM_2.5_ and blood methylation at cg05575921 (AHRR). The fact that other environmental factors would have also accounted for this association cannot be completely rolled out. However, the emphasis on PM_2.5_ is because it contains PAHs which are related to AHRR. Exposure to PAHs is believed to cause smoking-related AHRR methylation [9]. Moreover, PM_2.5_ is a serious problem in Taiwan and PM-related PAHs pose most of the health problems in humans [20].

## Conclusion

In conclusion, living in PM_2.5_ areas was inversely associated with blood AHRR methylation levels at cg05575921. Compared with Northern Taiwan (which has the lowest PM_2.5_ levels), living in North-Central, Central, and Southern Taiwan was associated with lower blood AHRR methylation levels at cg05575921. The methylation levels were lowest in those residing in the southern area (which has the highest PM_2.5_) levels followed by central and north-central areas. Moreover, PM_2.5_ was inversely associated with methylation levels at cg05575921 when it was included in the regression model. AHRR (cg05575921) methylation might serve as an indicator of differential exposures to PM_2.5_ and lung cancer which is also a PM_2.5_-related disease. Further investigations to confirm these findings are recommended.

## Methods

### Data source

Data used in the current study were obtained from the Taiwan Biobank which was founded in 2005. The biobank aims at undertaking large-scale cohort and case-control studies through the combination of genetic and medical information [48, 49]. The information collected is currently serving as one of the cornerstones of medical research in Taiwan. It is hoped that the identification of disease risk factors and the underlying mechanisms would lead to the development of better treatments and prevention strategies, hence, reduced medical costs [48, 49]. As a result, the health of the population would probably be promoted and improved.

Information about recruitment in the Taiwan Biobank project is obtained through brochures, posters, media, and websites. Interested volunteers are asked to provide their contact information. These volunteers are contacted to confirm their willingness to participate, and those who fulfill the recruitment requirements (strictly Taiwanese aged 30–70 years with no personal history of cancer) are assigned to specific recruitment centers. Currently, the biobank comprises 29 recruitment centers with each city or county having at least 1 center [49]. Before data are collected, all the participants sign a letter of consent.

### Data collection

Data were collected by trained medical researchers through questionnaires (e.g., residence, sex, age, smoking, alcohol drinking, exercise habits, and exposure to SHS), physical examination (e.g., weight and height), and blood examination (e.g., DNA methylation). Participants were considered as non-smokers if they reported to have never smoked or have not continuously smoked for at least 6 months. Former smokers were those who reported to have continuously smoked for at least 6 months but were currently not smoking. Current smokers were those who reported to have continuously smoked for at least 6 months and were currently smoking. Participants were considered non-drinkers if they did not drink or drank less than 150 cc of alcohol per week continuously for 6 months. Former drinkers were those who abstained from alcohol for more than 6 months while current drinkers were those who drank at least 150 cc of alcohol per week continuously for 6 months. Exercise habits were categorized under “yes” if participants reported a habit of exercising at least three times per week (each exercise time > 30 min) and under “no” if they did not exercise at least three times per week. Individuals considered to be exposed to SHS were those who reported being exposed to SHS for at least 5 minutes per hour. BMI was derived from weight and height as BMI = weight (kg)/height (m^2^).

DNA was extracted from whole blood using an automated extraction machine called chemagic^TM^ Prime^TM^ instrument. DNA length was checked using Fragment Analyzer (Agilent). Its quality was assessed using the ratio of absorbance at 260/280 with the purity index set as 1.6–2.0. The samples that passed quality control were stored at − 80 °C for long-term use. DNA methylation was determined using the Illumina Infinium MethylationEPIC BeadChip which has been previously described [50–52]. In brief, DNA samples were treated with sodium bisulfite conversion using the EZ DNA Methylation Kit (Zymo Research, CA, USA). Data quality control was performed based on the Illumina® GenomeStudio® Methylation Module v1.8 [53]. Samples with *P* value > 0.05 or bead count < 3 were removed. Dye bias across batches was adjusted by normalization and background correction was performed. Outliers were removed using the median absolute deviation method. The methylation level at each CpG site was determined using *β* values. The *β* values were derived using the formula *β* = *M*/(*M* + *U*), where *M* = methylated intensity and *U* = unmethylated intensity.

### Study participants

**Fig. 1:**
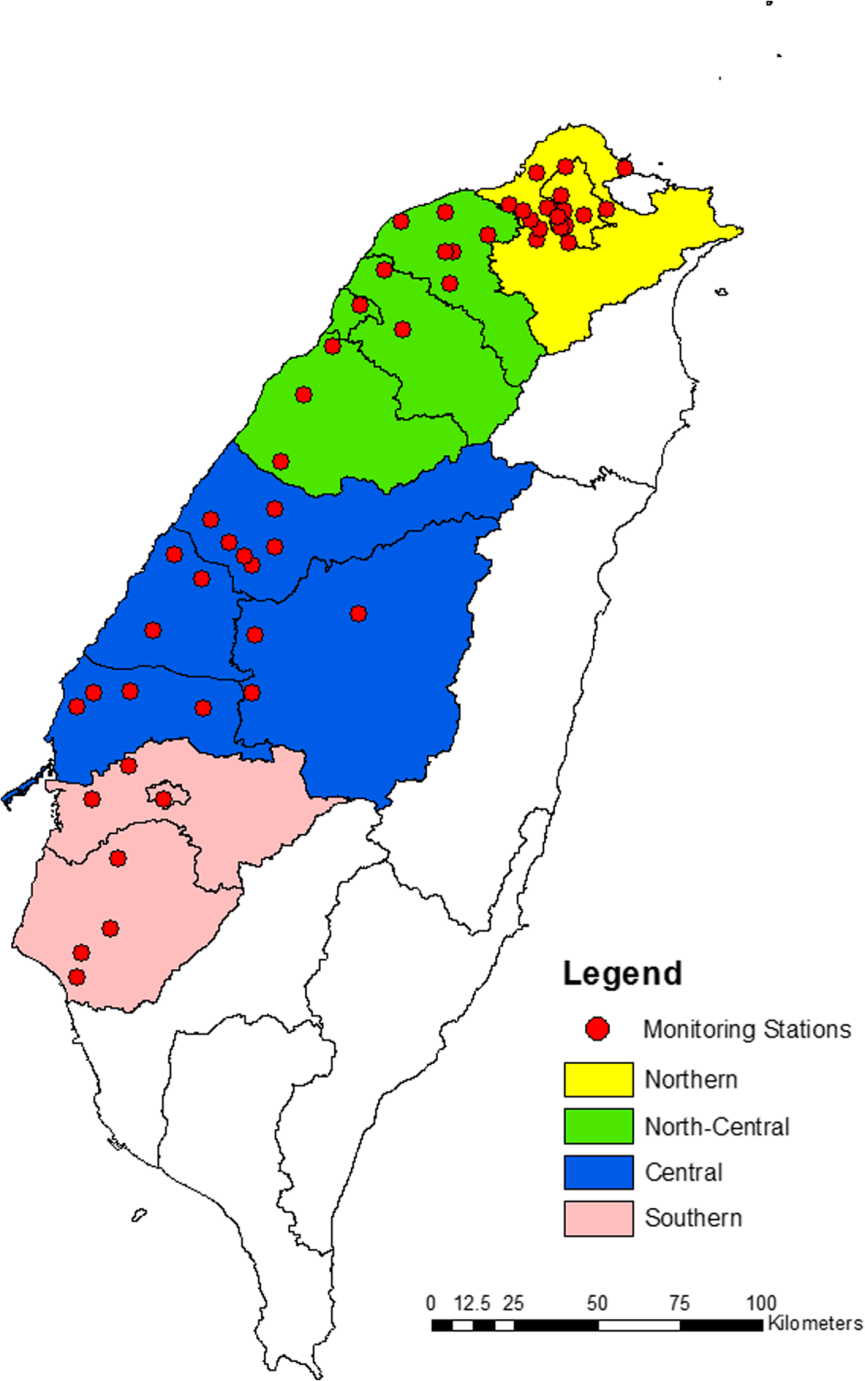
Map showing the study areas and the location of monitoring stations

Data used in the current study were retrieved from the Taiwan Biobank dataset (2008–2015). A total of 1142 individuals aged between 30 and 70 years with no personal history of cancer were initially enrolled. We excluded 301 current and former smokers, as well as 133 individuals who did not live in the study area (Fig. 1) from the study. The final sample included 708 non-smoking participants consisting of 279 men and 429 women.

The study area was categorized into Northern (Taipei and New Taipei Cities), North-Central (Taoyuan and Hsinchu Cities, Hsinchu, Miaoli, and Taoyuan Counties), Central (Taichung City, Nantou, Changhua, and Yunlin Counties), and Southern Taiwan (Chiayi County, Tainan, and Chiayi Cities). These areas are well known for varying PM_2.5_ levels. That is, PM_2.5_ levels increase as one moves from the north through the center towards the south of Taiwan [26–29]. Overall, there were 244, 121, 134, and 209 participants residing in the northern, north-central, central, and southern areas, respectively. The number of monitoring stations was 18 for the northern, 12 for the north-central, 16 for the central, and 7 for the southern area. In this study, we used the average PM_2.5_ levels (2006–2011) of the monitors of each area (northern, north-central, central, and southern areas) to estimate the PM_2.5_ exposure of participants. The location of participants and monitoring stations is shown in Fig. 1. The current study was approved by the Chung Shan Medical University Institutional Review Board (CS2-17070).

### Statistical analysis

Cell-type heterogeneity was corrected using the Reference-Free Adjustment for Cell-Type composition (ReFACTor) approach with the R software described by Rahmani and colleagues [54]. The association between blood methylation levels at cg05575921 and living in PM_2.5_ areas was determined using multiple linear regression analysis. Adjustments were made for sex, age, alcohol drinking, exercise, BMI, exposure to SHS, and cell type composition [54]. The inclusion of sex, age, alcohol drinking, exercise, and BMI as confounders is because they are common factors that have been associated with health. Moreover, DNA methylation predictors have been shown to correlate with these factors [55]. For instance, in previous studies, older age was significantly associated with lower cg05575921 methylation [24] and increased PM_2.5_-related mortality [56]. The male sex was significantly associated with lower cg05575921 methylation [24] while the female sex was significantly associated with an increased risk of PM_2.5_-related mortality [56]. The regression analysis was performed with the SAS 9.3 software (SAS Institute, Cary, NC).

## Additional files

Additional file 1: Spearman correlation between PM_2.5_ concentrations (μg/m^3^) and mean methylation levels (beta values) in the northern, north-central, central, and southern areas. The methylation beta values decrease as PM_2.5_ levels increase. (DOCX 40 kb)

Additional file 2: AHRR CpG sites significantly associated with PM_2.5._ (DOCX 20 kb)

## Abbreviations

AHRR: Aryl hydrocarbon receptor repressor
CpG: Cytosine-phosphate-guanine
DNA: Deoxyribonucleic acid
MOST: Ministry of Science and Technology
*n*: Sample size
PAH: Polycyclic aromatic hydrocarbon
PM_2.5_: Particulate matter < 2.5 microns in aerodynamic diameter
SD: Standard deviation
SHS: Second-hand smoke
*β*: Regression coefficient
