# Supplementary material for: Methylation at cg05575921 of a smoking-related gene (AHRR) in non-smoking Taiwanese adults residing in areas with different PM2.5 concentrations
Source: Clin Epigenetics. 2019 May 6;11:69. doi: 10.1186/s13148-019-0662-9 (PMC6503351; doi:10.1186/s13148-019-0662-9)
Supplement: Supplementary file 1 — Spearman correlation between PM2.5 concentrations (μg/m3) and mean methylation levels (beta values) in the northern, north-central, central, and southern areas. The methylation beta values decrease as PM2.5 levels increase. (DOCX 40 kb) [file 13148_2019_662_MOESM1_ESM.docx]

**Supplementary Figure 1. Spearman correlation between PM_2.5_ concentrations (μg/m^3^) and mean methylation levels (beta-values) in the Northern, North-Central, Central and Southern areas. The methylation beta-values decrease as PM_2.5_ levels increase.**
