# Supplementary material for: Methylation at cg05575921 of a smoking-related gene (AHRR) in non-smoking Taiwanese adults residing in areas with different PM2.5 concentrations
Source: Clin Epigenetics. 2019 May 6;11:69. doi: 10.1186/s13148-019-0662-9 (PMC6503351; doi:10.1186/s13148-019-0662-9)
Supplement: Supplementary file 2 — AHRR CpG sites significantly associated with PM2.5. (DOCX 20 kb) [file 13148_2019_662_MOESM2_ESM.docx]

**Supplementary Table 1. AHRR CpG sites significantly associated with PM_2.5_**

| CpG sites | Beta | P-value |
| --- | --- | --- |
| ncg05575921 | -0.00124 | <.0001** |
| ncg26703534 | -0.00127 | <.0001** |
| ncg25648203 | -0.00078 | <.0001** |
| ncg09634134 | 0.00103 | <.0001** |
| ncg09470163 | 0.00079 | <.0001** |
| ncg26487191 | 0.00072 | 0.0002** |
| ncg22356527 | 0.00070 | <.0001** |
| ncg09662430 | 0.00067 | <.0001** |
| ncg19405895 | -0.00034 | 0.0002** |
| ncg21813876 | -0.00034 | <.0001** |
| ncg17166056 | -0.00040 | <.0001** |
| ncg11445754 | -0.00041 | 0.0001** |
| ncg09078081 | -0.00042 | <.0001** |
| ncg09584122 | -0.00043 | <.0001** |
| ncg04939692 | -0.00049 | <.0001** |
| ncg11258653 | -0.00053 | <.0001** |
| ncg05842815 | -0.00054 | <.0001** |
| ncg09854184 | -0.00055 | <.0001** |
| ncg00629928 | -0.00060 | <.0001** |
| ncg07780979 | -0.00060 | <.0001** |
| ncg14982043 | -0.00061 | 0.0002** |
| ncg14684960 | -0.00064 | <.0001** |
| ncg17472786 | -0.00064 | 0.0002** |
| ncg11610050 | -0.00069 | <.0001** |
| ncg17310215 | -0.00070 | <.0001** |
| ncg16219322 | -0.00070 | <.0001** |
| ncg05655106 | -0.00070 | <.0001** |
| ncg14807090 | -0.00070 | <.0001** |
| ncg08606254 | -0.00072 | <.0001** |
| ncg16049691 | -0.00074 | <.0001** |
| ncg05934812 | -0.00074 | <.0001** |
| ncg24688690 | -0.00075 | <.0001** |
| ncg11557553 | -0.00076 | <.0001** |
| ncg18205372 | -0.00081 | <.0001** |
| ncg06047773 | -0.00086 | <.0001** |
| ncg01970407 | -0.00094 | <.0001** |
| ncg26850624 | -0.00099 | <.0001** |
| ncg17287155 | -0.00103 | <.0001** |
| ncg04551776 | -0.00108 | <.0001** |
| ncg16336872 | -0.00113 | <.0001** |
| ncg26529655 | -0.00126 | <.0001** |
| ncg07137034 | -0.00129 | 0.0002** |
| ncg09078014 | -0.00153 | <.0001** |
| ncg13707777 | -0.00161 | <.0001** |
| ncg04202140 | -0.00175 | <.0001** |
| ncg09874656 | -0.00058 | 0.0345* |
| ncg24256039 | 0.00091 | 0.0262* |
| ncg05758931 | 0.00085 | 0.0101* |
| ncg05527650 | 0.00072 | 0.0042* |
| ncg04135110 | 0.00066 | 0.0173* |
| ncg02385153 | 0.00059 | 0.0261* |
| ncg22698028 | 0.00051 | 0.0035* |
| ncg26954197 | 0.00046 | 0.0034* |
| ncg14453201 | 0.00042 | 0.0418* |
| ncg11902777 | 0.00042 | 0.0494* |
| ncg08714121 | 0.00033 | 0.0046* |
| ncg16577724 | 0.00021 | 0.0208* |
| ncg05601199 | -0.00012 | 0.0246* |
| ncg22816059 | -0.00022 | 0.0388* |
| ncg22030839 | -0.00023 | 0.0039* |
| ncg02356223 | -0.00029 | 0.0076* |
| ncg14448919 | -0.00029 | 0.0363* |
| ncg11148817 | -0.00029 | 0.0035* |
| ncg21144161 | -0.00030 | 0.0394* |
| ncg02088390 | -0.00031 | 0.0127* |
| ncg03569073 | -0.00033 | 0.0037* |
| ncg06605558 | -0.00034 | 0.003* |
| ncg24130459 | -0.00037 | 0.0044* |
| ncg11763982 | -0.00038 | 0.0339* |
| ncg19039843 | -0.00039 | 0.0087* |
| ncg14219121 | -0.00041 | 0.0020* |
| ncg04066994 | -0.00041 | 0.0052* |
| ncg27240182 | -0.00042 | 0.0003* |
| ncg12845747 | -0.00043 | 0.0064* |
| ncg22937882 | -0.00043 | 0.0015* |
| ncg13404472 | -0.00046 | 0.0012* |
| ncg21161138 | -0.00046 | 0.0068* |
| ncg12806681 | -0.00047 | 0.0021* |
| ncg03604011 | -0.00047 | 0.0014* |
| ncg00300637 | -0.00052 | 0.0199* |
| ncg21972741 | -0.00053 | 0.0011* |
| ncg21880882 | -0.00060 | 0.0005* |
| ncg15945600 | -0.00061 | 0.0151* |
| ncg07448928 | -0.00066 | 0.0009* |
| ncg23576855 | -0.00085 | 0.0324* |
| ncg24980413 | -0.00095 | 0.0090* |
| ncg09478603 | -0.00099 | 0.0254* |
| ncg00976097 | -0.00102 | 0.0041* |
| ncg18615970 | -0.00110 | 0.0007* |
| ncg12937950 | -0.00125 | 0.0011* |
| ncg26076054 | -0.00159 | 0.0261* |
| ncg08238319 | 0.00229 | 0.2848 |
| ncg04369835 | 0.00098 | 0.0918 |
| ncg06802630 | 0.00073 | 0.1741 |
| ncg14714797 | 0.00050 | 0.207 |
| ncg07943658 | 0.00044 | 0.0593 |
| ncg01899089 | 0.00029 | 0.3683 |
| ncg12202185 | 0.00028 | 0.3681 |
| ncg00699559 | 0.00024 | 0.1647 |
| ncg11554391 | 0.00023 | 0.2004 |
| ncg16896326 | 0.00023 | 0.0627 |
| ncg10841124 | 0.00023 | 0.3482 |
| ncg23916896 | 0.00022 | 0.6001 |
| ncg04141806 | 0.00021 | 0.1581 |
| ncg26987759 | 0.00017 | 0.6301 |
| ncg09454315 | 0.00015 | 0.7834 |
| ncg04879308 | 0.00012 | 0.7949 |
| ncg09338136 | 0.00012 | 0.5637 |
| ncg19772705 | 0.00012 | 0.331 |
| ncg19442702 | 0.00012 | 0.3155 |
| ncg02527419 | 0.00012 | 0.2679 |
| ncg01958142 | 0.00010 | 0.5237 |
| ncg20344367 | 0.00009 | 0.4511 |
| ncg16501378 | 0.00009 | 0.4213 |
| ncg13023972 | 0.00008 | 0.6807 |
| ncg08519949 | 0.00008 | 0.3134 |
| ncg10869925 | 0.00007 | 0.5715 |
| ncg03561637 | 0.00003 | 0.8113 |
| ncg06036945 | 0.00003 | 0.6117 |
| ncg16995193 | 0.00002 | 0.9051 |
| ncg01571467 | 0.00001 | 0.9245 |
| ncg18541609 | 0.00001 | 0.9071 |
| ncg04286878 | 0.00001 | 0.8182 |
| ncg05516328 | 0.00001 | 0.9915 |
| ncg04023872 | 0.00000 | 0.947 |
| ncg14690983 | 0.00000 | 0.9886 |
| ncg16325394 | 0.00000 | 0.9978 |
| ncg14647125 | 0.00000 | 0.9998 |
| ncg22103736 | 0.00000 | 0.9989 |
| ncg17386114 | -0.00001 | 0.9059 |
| ncg08802770 | -0.00001 | 0.7819 |
| ncg00401753 | -0.00002 | 0.8902 |
| ncg15168497 | -0.00003 | 0.9375 |
| ncg04021706 | -0.00004 | 0.7769 |
| ncg06678548 | -0.00004 | 0.7905 |
| ncg15179499 | -0.00004 | 0.8168 |
| ncg06035956 | -0.00004 | 0.6756 |
| ncg18584368 | -0.00005 | 0.6193 |
| ncg17668415 | -0.00007 | 0.7485 |
| ncg14454127 | -0.00007 | 0.6013 |
| ncg26320890 | -0.00008 | 0.7341 |
| ncg17989581 | -0.00008 | 0.1541 |
| ncg17248487 | -0.00010 | 0.5917 |
| ncg16294152 | -0.00010 | 0.7196 |
| ncg24081180 | -0.00010 | 0.4756 |
| ncg08491376 | -0.00012 | 0.3129 |
| ncg20433154 | -0.00013 | 0.4458 |
| ncg25430111 | -0.00013 | 0.1744 |
| ncg20310920 | -0.00013 | 0.6137 |
| ncg13275321 | -0.00013 | 0.4915 |
| ncg16371648 | -0.00015 | 0.6605 |
| ncg08902828 | -0.00015 | 0.4867 |
| ncg24891125 | -0.00016 | 0.3334 |
| ncg03491025 | -0.00016 | 0.2049 |
| ncg01141993 | -0.00017 | 0.1356 |
| ncg11827403 | -0.00018 | 0.1552 |
| ncg08858540 | -0.00018 | 0.3143 |
| ncg12207033 | -0.00018 | 0.1887 |
| ncg20554397 | -0.00022 | 0.0802 |
| ncg11894422 | -0.00022 | 0.1394 |
| ncg09084391 | -0.00022 | 0.2248 |
| ncg16172278 | -0.00023 | 0.0754 |
| ncg03891523 | -0.00024 | 0.1391 |
| ncg14744022 | -0.00024 | 0.1056 |
| ncg00731338 | -0.00024 | 0.5354 |
| ncg01097768 | -0.00027 | 0.0917 |
| ncg16189952 | -0.00028 | 0.2359 |
| ncg27464615 | -0.00031 | 0.0690 |
| ncg21623028 | -0.00032 | 0.0935 |
| ncg05597431 | -0.00038 | 0.0506 |
| ncg12961784 | -0.00039 | 0.2992 |
| ncg24090911 | -0.00041 | 0.0645 |
| ncg07599136 | -0.00174 | 0.2389 |
| ncg24955955 | -0.00196 | 0.1947 |
| ncg08916839 | -0.00242 | 0.1276 |
| ncg16081854 | -0.00297 | 0.0582 |

* = P < 0.05, ** = P < 0.0002841 (Bonferoni significance)
